# What about “Pharming”? Issues Regarding the Misuse of Prescription and Over-the-Counter Drugs

**DOI:** 10.3390/brainsci10100736

**Published:** 2020-10-14

**Authors:** Stefania Chiappini, Fabrizio Schifano

**Affiliations:** Psychopharmacology, Drug Misuse and Novel Psychoactive Substances Research Unit, School of Life and Medical Sciences, University of Hertfordshire, Hertfordshire AL10 9AB, UK; stefaniachiappini9@gmail.com

**Keywords:** drug diversion, drug misuse, drug abuse, non-medical drug use, pharmacovigilance, prescribing drugs’ misuse, over-the-counter drugs’ misuse, novel psychoactive substances

## Abstract

Recently, a range of prescription and over-the-counter (OTC) drugs have emerged as being used recreationally, either on their own or in combination with other substances, both licit and illicit, including new psychoactive substances (NPS). Among them, the misuse of prescription drugs involves not only traditionally recorded substances, such as benzodiazepines and opioid pain relievers, but also gabapentinoids (e.g., pregabalin and gabapentin); some antidepressants, e.g., bupropion and venlafaxine; some second-generation antipsychotics, e.g., quetiapine and olanzapine. Moreover, the use of some OTC for recreational purposes appears on the increase, especially in vulnerable categories such as young people/youths, including the use of high dosages of the antidiarrheal loperamide; first-generation antihistamines, e.g., promethazine, cyclizine, and diphenhydramine; cough and cold preparations containing dextromethorphan and/or codeine. In this context, the role of the Internet has rapidly increased, playing a significant role both in the diffusion of emerging trends of drug misuse among users and experimenters, and the marketing, sale, and distribution of drugs through online pharmacies. This phenomenon within the context of a rapidly modifying drug scenario is a globally recognized health problem, determining severe adverse consequences, including fatalities, and represents a challenge for clinicians in general, psychiatrists, public health, and drug-control policies.

Over the last twenty years or so, both the emerging use of new psychoactive substances (NPS) and the misuse of medications that are not already controlled are rapidly becoming a worldwide health concern [1,2,3,4]. With the term “misuse”, we refer here to the intentional and inappropriate use of a product other than as prescribed or not in accordance with the authorized product information; at times, this is carried out for a perceived reward, including “getting high” and euphoria (for a thorough overview of the issue, see Schifano, 2020 [5]). Prescription and over-the-counter (OTC) medication misuse is being perceived to be a significantly under-recognized issue affecting a range of vulnerable individuals [6]. However, according to data from National Surveys on Drug Use and Health in the US, the intentional misuse of prescribed and OTC medications has climbed steadily [1,7]. Moreover, medications’ misuse appeared to be second only to marijuana use as the nation’s most common type of drug use, specifically driven by the already recorded rise in the opioid use, benzodiazepines, and prescription stimulants, e.g., amphetamines, methamphetamines, methylphenidate [7,8]. In Europe, data from surveys; poison control center records; and pharmacovigilance studies have suggested increasing levels of the so-called “pharming”. This a phenomenon involving the non-medical use and misuse of prescription and OTC medications, including gabapentinoids; some antipsychotics, e.g., quetiapine; some antidepressants, e.g., venlafaxine and bupropion; pain relievers, e.g., opioids; z-hypnotics; loperamide; antihistamines, e.g., chlorpheniramine, cyclizine, and diphenhydramine; some anticholinergics, e.g., dimenhydrinate; and cough and cold preparations, particularly those containing promethazine, codeine, and/or dextromethorphan [1,5,7]. Due to their high and super-high dosage intake [5,9] and their use in combination with alcohol and remaining recreational drugs, “pharming” may also cause significant morbidity [1]. Indeed, the non-medical use of medications may result in serious clinical effects with potential life-threatening complications, drug-seeking behavior, dependence, and the occurrence of related withdrawal syndrome as well [1,7]. “Pharming” may be facilitated by medications’ relatively easy accessibility; low cost; decreased perception of potential for harm; and growing social acceptance or less stigmatization [1,7,8,10]. Misusing prescription and OTC drugs may well be solicited by the purpose for which the medication was firstly prescribed, e.g., pain relievers or tranquilizers used at high dosages, respectively, to manage physical pain or to help sleep. Conversely, they may also be consumed for recreational purposes; to manage withdrawal symptoms; to feel good and get “high”; or to relax and relieve tension associated with emotional or psychiatric distress [7,8,11]. Similar to what happened in the past with benzodiazepines and z-drugs, where pre-marketing procedures were unable to appropriately identify the misuse and diversion potential of some drugs [12], the misusing liability of some of the medications that are currently part of the “pharming” phenomenon is likely to fully emerge over time. This better understanding of the phenomenon will occur through further effective post-marketing surveillance (PMS) studies, e.g., through the monitoring of adverse drug reactions (ADRs) and other pharmacovigilance approaches [13,14,15,16]. As such, PMS may occur through national reporting schemes (e.g., the Yellow Card Scheme of the Medicines and Healthcare products Regulatory Agency (MHRA) in the United Kingdom [17]; and the European Medicines Agency (EMA) EudraVigilance system [18]) and active post-marketing surveys [12]. Data from pharmacovigilance studies seem to suggest that specific drug misuse liability levels may be associated with idiosyncratic reactions; use of ultra-high dosages; drug interactions with licit and illicit substances; and other organic illnesses [2,5,19]. Indeed, the lack of mention and information on the misuse potential of an index medication leaflet does not necessarily mean that the specific molecule does not actually produce these effects.

In this context, the role of the Internet in changing drug misuse scenarios is central, since playing a significant role in the marketing, sale, and distribution of a range of psychoactive, including prescription, drugs [5,20,21]. Indeed, online drug forum communities and social networks are popular with some educated or informed users [5], known as “psychonauts” [9], who typically “experiment” with a range of psychotropics, including prescribed drugs, and then share information with peers. In parallel with this, access to online pharmacies to purchase medicinal compounds has increased over the last 10 years or so [22].

This ever-changing drug scenario represents a challenge for accident and emergency units; healthcare services [19]; public health; psychiatry; and drug-control policies [23]. Indeed, in case of some drug intoxication presentations, drug detection for some of those medications that are central to the “pharming” phenomenon (e.g., loperamide; antidepressants; antipsychotics; cough and cold preparations; anti-histamines, etc.) may be difficult because they cannot be detected by standard forensic drug tests [7]. Hence, a multicomponent approach is here recommended; this approach should include monitoring of drug utilization; tracking users’ posts on social media; and exploration of healthcare databases. Finally, appropriate and specific clinical guidelines for the treatment of misuse of prescription and OTC drugs should be promoted and implemented [14,24].
